# High Quality *Aspergillus aculeatus* Genomes and Transcriptomes: A Platform for Cellulase Activity Optimization Toward Industrial Applications

**DOI:** 10.3389/fbioe.2020.607176

**Published:** 2021-01-27

**Authors:** Wuttichai Mhuantong, Salisa Charoensri, Aphisit Poonsrisawat, Wirulda Pootakham, Sithichoke Tangphatsornruang, Chatuphon Siamphan, Surisa Suwannarangsee, Lily Eurwilaichitr, Verawat Champreda, Varodom Charoensawan, Duriya Chantasingh

**Affiliations:** ^1^National Center for Genetic Engineering and Biotechnology, Thailand Science Park, Pathum Thani, Thailand; ^2^National Omics Center, National Science and Technology Development Agency, Thailand Science Park, Pathum Thani, Thailand; ^3^Department of Biochemistry, Faculty of Science, Mahidol University, Bangkok, Thailand; ^4^Integrative Computational Bioscience Center, Mahidol University, Nakhon Pathom, Thailand; ^5^Systems Biology of Diseases Research Unit, Faculty of Science, Mahidol University, Bangkok, Thailand

**Keywords:** plant biomass degradation, cellulolytic enzyme activities, genome sequencing, transcriptome sequencing, *de novo* assembly

## Introduction

Plant biomass is an important feedstock for the production of biofuel and other value-added chemicals in biorefinery industry (Rosales-Calderon and Arantes, 2019). In theory, a ton of cellulose could be converted to ~170 gallons of bioethanol, and thus the global cellulosic biomasses (including corn stover, rice straw and wood residues) can potentially be used to produce over 167 billion gallons of bioethanol annually (Murdock et al., 2019). Despite its great potential, the challenges of biomass utilization lie in the current uncompetitive production cost when compared to that of fossil fuel. Feedstock prices, raw material transportation, and production process, especially pretreatment, saccharification, and fermentation, are major factors of the high capital costs in the production of biofuels (Lynd et al., 2008; Mithra and Padmaja, 2017; Zoghlami and Paës, 2019). Unlike other bottlenecks, the pretreatment and saccharification processes can be biologically catalyzed by enzymes, especially from microorganisms, providing one of the most environmentally and economically sustainable solutions (Wang and Sun, 2010; Wang et al., 2012). Evidently, it has already been shown that genetic modification of a cellulase producing microbe, *Trichoderma reesei*, could provide a low-cost on-site enzyme production in Brazil (Zhuang et al., 2007), where the genetically engineered *T. reesei* could efficiently utilize local biomasses such as soybean hulls and molasse.

Cellulolytic enzymes from filamentous fungi such as *Aspergillus* (de Vries et al., 2000, 2017), *Neurospora* (Campos Antoniêto et al., 2017), *Penicillium* (Gusakov and Sinitsyn, 2012), and *Trichoderma* (Novy et al., 2019) have been screened for cellulose-, hemicellulose-, and other polysaccharide-degrading enzyme activities, and successfully applied in lignocellulose conversion. Among the cellulase-producing fungi, *Aspergillus aculeatus* is one of the most important models for optimization of cellulolytic enzymes due to its versatile metabolism and ability to grow on a wide range of substrates and under various conditions (Jayani et al., 2005; Poonsrisawat et al., 2014; Kunitake et al., 2015; Zhao et al., 2020). Different types of enzymes from *A. aculeatus* have already been used effectively by us and others, in the productions of animal feed (Saxena et al., 2019), paper (Jayani et al., 2005), cotton fabric (Abdulrachman et al., 2017), and bioethanol (Poonsrisawat et al., 2014). To explore new microorganisms for industrial applications, we have previously screened over 1,300 isolates from our institutional depository known as “Thailand Bioresource Research Center (TBRC, www.tbrcnetwork.org),” and the fungus *A. aculeatus* strain “BCC199” (also known as TBRC277 in our repository) came out as one of the most promising cellulolytic enzyme producers in our collection, based on its cellulolytic enzyme activity on saccharification of different types of biomass (Suwannarangsee et al., 2012, 2014). Random mutagenesis had already been applied for further industrial development (Champreda et al., 2019), leading to an increment of cellulolytic activity up to 2-folds. However, such strategies are time-consuming and not yet able to sufficiently ameliorate the enzyme activity to the level that is economically feasible for industrial production and utilization.

Here, we have sequenced and comprehensively analyzed the genomes and transcriptomes of *A. aculeatus* strain “BCC199” (TBRC277) and two additional strains representing different levels of cellulase activities. The three genomes were sequenced and assembled at higher coverages, as compared to the *A. aculeatus* genomes publicly available. Transcriptome of each strain were also constructed to facilitate transcript annotation. Both types of omic data sets not only provide the highest quality reference genomes and annotations of the cellulolytic enzyme-producing fungus *A. aculeatus*, which can be used to investigate distinct genomic features of the fungus that may play the role in cellulolytic activity.

## Data

The genomes of three *A. aculeatus* strains with different cellulase enzyme activities (see Table 1—Enzymatic activities, and Methods), including BCC56535 (the strain representing “low” cellulase activity), BCC199 (representing “moderate” activity), and HUT2365 (representing “high” activity), were completely sequenced in-house using the PacBio RS II platform (Pacific Biosciences, USA), giving the total circular consensus sequences of 345.3–463.3 Mb, equivalent to the genome coverages of 9.3X-12.1X (Table 1—Genome sequencing and analyses, see Supplementary Figure 1 for the analytical process). The final genome sizes after assembly were between 36.0 and 37.7 Mb, similar to the size of the most closely-related publicly available genome *A. aculeatus* ATCC16872 (36.01 Mb) (https://mycocosm.jgi.doe.gov/Aspac1/Aspac1.home.html), and with consistent GC contents of 49.4–50.8%.

**Table 1:** Key features of three *A. aculeatus* strains, their genomes, and transcriptomes.

**Enzyme activities (unit/mg of protein)**	**BCC56535**	**BCC199**	**HUT2365**
FPase	0.009^c^ ± 0.003	0.039^b^ ± 0.007	0.327^a^ ± 0.040
CMCase	0.563^c^ ± 0.045	2.879^b^ ± 0.496	10.573^a^ ± 0.619
**Genome sequencing and analyses**	**BCC56535**	**BCC199**	**HUT2365**
Sequence base (CCS)	345,347,605	463,390,377	423,885,346
Estimated genome coverage^*^	9.3X	12.1X	11.4X
Assembled genome size (bp)	37,193,364	37,670,955	36,004,422
No. contigs (>=50 kb)	34	11	14
N50	1,914,919	4,861,851	3,415,456
L50	7	4	4
GC (%)	49.4	49.5	50.8
**Transcriptome sequencing and analyses**	**BCC56535**	**BCC199**	**HUT2365**
Average no. of raw reads (reads)	47.69 M	47.31 M	71.61 M
Average no. of clean reads (reads)	46.97 M	45.71 M	70.18 M
Sequence depth	119.79	116.75	136.78
Q20 (%)	97.21	96.26	97.08
GC (%)	55.70	55.66	54.67
No. predicted gene	12,005	11,442	11,489

*Enzymatic assays were performed in triplicate, and the data were shown as means ± standard deviations. The data were compared by ANOVA test. Means in a roll followed by different letters (a–c) are significant at p < 0.05. Data were analyzed separately for each enzymatic activity. HUT, Hiroshima University Culture Collection; BCC, BIOTEC Culture Collection; CCS, Circular Consensus Sequence with corrections*.

* *Sequenced base (bp)/estimated genome size (A. aculeatus ATCC16872, 36.01 Mb, https://mycocosm.jgi.doe.gov/Aspac1/Aspac1.home.html)*.

To improve the annotation and functional analyses of the newly sequenced *A. aculeatus* genomes, we constructed multiple reference transcriptomes of each strain using RNA-seq, and obtained the averages of 47.69, 47.31, and 71.61 M clean reads from BCC56535, BCC199, and HUT2365, respectively (Table 1 - Transcriptome sequencing and Supplementary Table 3). *De novo* transcriptome assembly followed by integrating the gene models with those from genome annotation (see Methods and Supplementary Figure 1), yielded comparable genomic features among the three *A. aculeatus* genomes, including number of genes (11,000–12,000 genes), GC contents, as well as genomic and functional contents, when compared against publicly available databases such as NCBI's non-redundant database (Coordinators, 2014), enzyme commission (Webb, 1990), and Gene Ontology (Carbon et al., 2009) (Supplementary Table 2). Two of the newly sequenced genomes, BCC199 and HUT2365, are of higher quality than the closely-related reference genomes publicly available, *A. aculeatus* ATCC16872 and *A. niger* CBS513.88, in terms of the largest contig sizes and N50 (Supplementary Figure 2).

**Figure 1:**
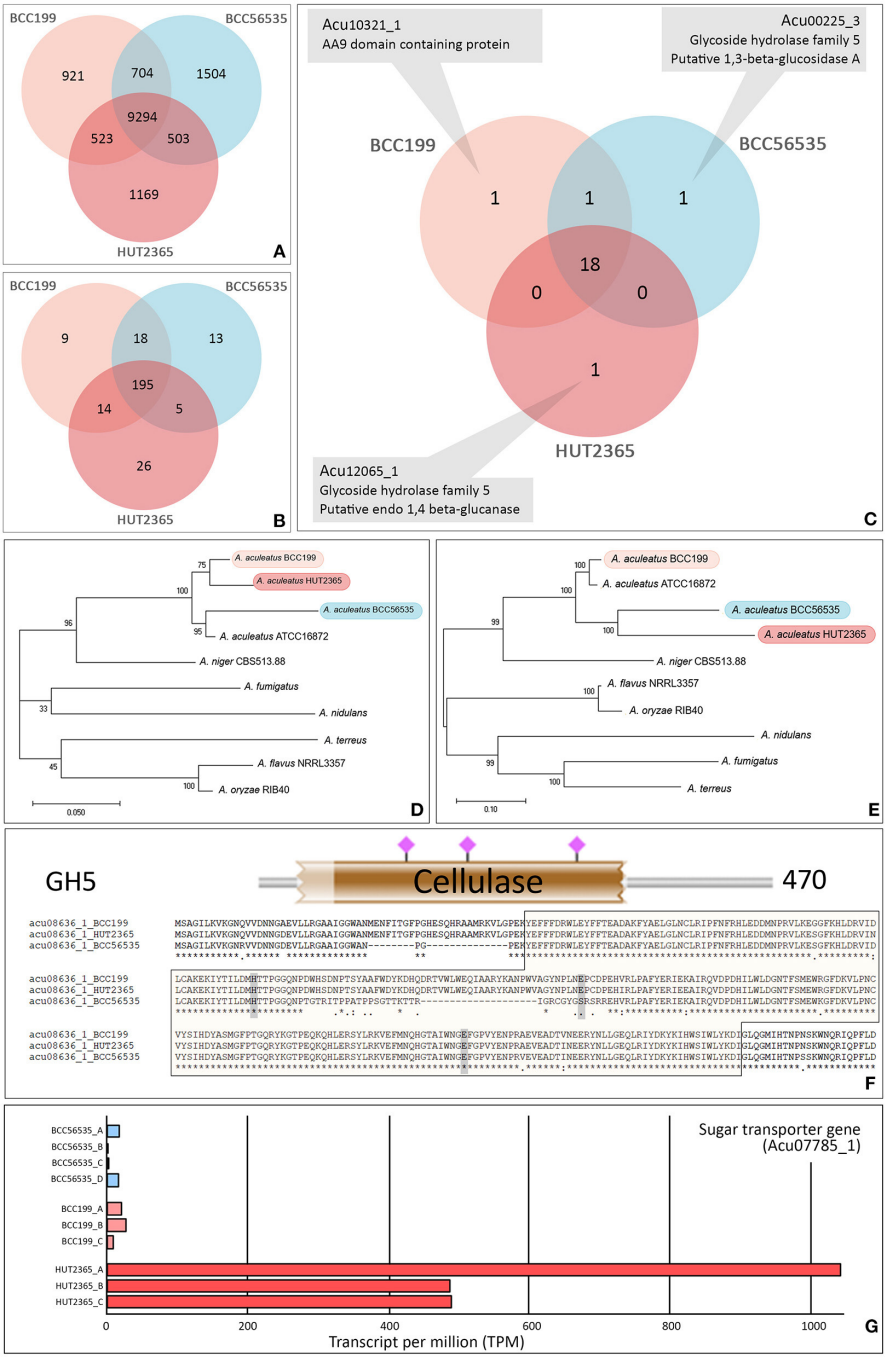
Gene annotation and prediction of biological function. Venn diagram showing the core orthologous and unique genes of the model *A. aculeatus* strains. **(A)** All predicted genes, **(B)** Glycoside Hydrolase (GH) families, and **(C)** Cellulose-degrading enzymes (GH5, 6, 7 and AA9 families). The genome of each strain possesses a unique putative gene: endo-1,4- glucanase gene (Acu12065_1) in HUT2365, 1,3-beta-glucosidase gene (Acu00225_3) in BCC56535, and AA9 domain-containing gene (Acu10321_1) in BCC199. **(D,E)** Phylogenetic relationships between studied *A. aculeatus* strains and other seven Aspergilli. Maximum likelihood phylogeny was constructed by using **(D)** the concatenation of housekeeping genes, translation elongation factor 1 alpha, ubiquitin-conjugating enzyme, and beta-tubulin, and **(E)** the concatenation of cellulose-degrading enzymes, GH5, 6, 7, and AA9. The percentages of reproducible trees in which the associated taxa clustered together in the bootstrap test (from 1,000 replicates) are shown next to the branches. **(F)** Sequence variations between the strains—e.g., multiple alignments, SNP positions in relation to functional genomic positions of glycoside hydrolase protein domain (in brown block), and active sites (shown by pink), predicted by HMMER 3.0 (Potter et al., 2018). **(G)** Example of a differentially expressed gene, a putative sugar transporter (Acu07785_1), which was uniquely over-expressed in all three replicates of HUT2365 transcriptomes.

We identified 14,618 orthologs and singletons and the vast majority (~97% in each strain) could be assigned with at least one known function based on the Uniport Blast hit (Supplementary Figure 3). Among these predicted genes, 9,294 (64%) have orthologs in all three strains (Figure 1A). Between 417–429 predicted genes (3.5–4%) of each strain were classified into one of the carbohydrate-active enzyme families (CAZy; Cantarel et al., 2009) (Supplementary Table 2); whereas the total of 280 genes were assigned as Glycosyl Hydrolase (GH) genes and 195 (~70%) of these are common in our three strains (Figure 1B). Focusing on the cellulase-relating genes (comprising GH5, 6, 7, and AA9 families), the vast majority (18 out 22 genes with these GH or AA9 functions, ~82%) are commonly found in the three strains (Figure 1C). Importantly, none of these families is uniquely present or absent in any particular strain (Supplementary Table 4). However, we noted that one cellulose-degrading gene, endo-1,4-glucanase gene (Acu12065_1: GH5), was exclusively found in the *A. aculeatus* HUT2365 (Figure 1C) and potentially be one of the candidate genes contributing to the higher cellulase activity in *A. aculeatus* HUT2365. The Acu12065_1 gene contains a signal peptide (1–17 amino acids), GH5 protein domain (58–245 amino acids), and conserved catalytic domain (157–166 amino acids) with one active site, E164 (Supplementary Figure 4).

We further investigated the phylogenetic relationships of the cellulose-degrading enzyme sequences (GH5, 6, 7, and AA9, Figure 1E), compared to those of selected housekeeping genes (Figure 1D). All three *A. aculeatus* strains and the publicly available ATCC16872 are closely related based on the housekeeping references, although BCC199 and HUT2365 appeared slightly closer to one another (Figure 1D). However, based on the cellulose-degrading protein sequences, our moderate-cellulase-activity BCC199 strain diverges the most among the three, and thus there is no clear evidence that the high cellulase activity seen in HUT2365 can be accounted for by the overall divergence of the protein sequences (Figure 1E). On the contrary, we observed several single nucleotide polymorphisms (SNPs) within the protein domains and some are in proximity to the active sites (Figure 1F, Supplementary Figures 5, 6), suggesting that small genomic variations such as point mutations in the cellulolytic genes between the strains might also play a role in their different cellulase activities.

In addition to the genomic data, our transcriptomics of the three *A. aculeatus* strains cultivated on plant biomass as the sole carbon source, can be used to investigate the expression of cellulolytic genes and their facilitating genes, that may contribute to the cellulose-degrading activities. Figure 1G provides an example of a highly expressed putative sugar transporter (Acu07785_1), which is unique to HUT2365. Indeed, we observed this and other Major Facilitator Superfamily (MFS) genes (Nogueira et al., 2020) being transcribed at higher levels in the high-cellulase-activity HUT2365 than in the other strains. As shown in *Neurospora crassa* (Wang et al., 2017) and *T. reesei* (Zhang et al., 2013), modifications of genes encoding glucose transporters greatly affected the cellulase production. These newly constructed transcriptomic data, together with the genomes and gene models described above, not only provide an important resource for genetic engineering of the fungal strains for industrial applications, but also serve as high-quality references for future characterization of the fungi and other related species and their genomic evolution.

## Materials and Methods

### Experimental Plan and Sample Preparation

The three *A. aculeatus* strains of interest: BCC199, BCC56535, and HUT2365, were cultivated on potato dextrose agar (PDA; Difco Laboratories, USA) at 25°C for 4 days, before spore collection. The spores were harvested by washing PDA plate with 10 ml of sterile water containing 1% Triton X-100 (Fischer Scientific, USA). In order to express cellulases (endoglucanases, exoglucanases, and β-D-glucosidases), the fungi have to be cultivated in a medium containing cellulose fiber, such as wheat bran and soybean. Therefore, fungal spores were then cultivated into 50 ml of wheat bran (3% w/v) and soybean (2% w/v) medium, to the final concentration of 10^7^ per ml. The fungal strains were cultured for 72 h in an aerobic cultivation condition at 30°C with 150 rpm in Innova 44R (Eppendorf AG, Germany). To collect cells and crude enzymes in the broth, fungal cells were separated from the whole broth by centrifugation (12,000 × g). The resulting supernatant was used in enzymatic assay. The cells were kept at −80°C for further genomic DNA and RNA isolations.

Total cellulase activity was measured with No. 1 Whatman filter paper (FPase activity) (Miller et al., 1960). For the major cellulose polymer degradation, endoglucanase activity was measured with carboxymethylcellulose (CMCase activity) (Miller et al., 1960). Briefly, the reaction mixture contained 20 uL of crude supernatant, 30 uL of 100 mM (pH 5.5) sodium acetate buffer and 50 uL of substrate to the 0.5% (w/v) final concentration in the final volume of 1 ml. The reaction was incubated for 60 min (for FPase assay) and for 10 min (for CMCase assay). The reaction was stopped by adding 100 uL of DNS. The enzymatic activities were calculated based on a corresponding standard containing glucose. One unit (U) of enzyme activity was defined as the amount of enzyme needed to liberate 1 umol of reducing sugars per minute. All samples were analyzed in triplicates and the mean values were calculated and reported.

### Genome Sequencing and Assembly

Genomic DNA (gDNA) samples of the three fungal strains grown in potato dextrose broth (PDB) were extracted using Wizard® genomic DNA purification kit (Promega, USA). The extracted gDNA was quality-checked using NanoDrop spectrophotometer (Thermo Scientific, USA) and agarose gel electrophoresis (Al Samarrai and Schmid, 2000). Purified gDNA was used to construct the DNA sequencing libraries, according to the PacBio RSII protocol (Pacific Bioscience, USA). Genome assembly was done using the SMRT Analysis Software version 2.3.0 (http://www.pacb.com/productsand-services/analytical-software/smrt-analysis/) with the following steps (Supplementary Figure 1): extracting subreads by bash5tools (https://github.com/PacificBiosciences/pbh5tools) with the default parameters [min read length = 500 bp, minimum polymerase read quality (QV) = 0.8]; correcting subreads by HGAP3 (Chin et al., 2013) with seed coverage = 30X; performing *de novo* assembly using HGAP3, with assembly target coverage = 40X. The assembled genomes were further polished by Quiver (https://github.com/PacificBiosciences/GenomicConsensus), using the default parameters (max divergence percentage = 30, minimum anchor size = 12). The contiguity of assembled sequences was evaluated by comparing the newly assembled genomes to those of publicly available *A. niger* CBS513.88 and *A. aculeatus* ATCC16872, using QUAST (Gurevich et al., 2013). The completeness of genome assembly and annotation was also assessed using the Benchmarking Universal Single-Copy Orthologs (BUSCO, version 4.1.2) pipeline (Simão et al., 2015), using the “Fungal set” (odb10) in the OrthoDB database (Zdobnov et al., 2017) as a reference. AUGUSTUS (Stanke et al., 2006), a Hidden Markov Model (HMM)-based gene prediction program, was employed to predict and locate the positions of genes (including start-stop codons, exons, and introns) in the three fungal genomes sequenced. *A. terreus* was used as the pre-trained model, as it was the most closely related reference genome available in the database.

### RNA-seq Library Preparation and Transcriptome Assembly

In addition to genome sequencing, we also analyzed the transcriptomes of the three *A. aculeatus* strains to improve the gene annotation. Fungal mycelium, cultured for 72 h in an aerobic cultivation condition at 30°C, was used to extract RNA following the Tri Reagent® protocol (MRC, USA). On-column DNase digestion kit (Qiagen, Germany) was used to further remove DNA traces. Then, the RNA was subjected to quantity and quality assessments by Advances Analyzer™ automated CS system (Advanced Analytical Technologies Inc., USA).

RNA-seq library preparation and sequencing were performed by Novogene (Beijing, China). In short, ribosomal RNA was degraded from 10 μg of total RNA using the Ribominus™ Eukaryotic kit (Life Technologies, USA). The RNA was enriched using the Absolutely mRNA Purification kit (Agilent Technologies, USA) and further quantified by BioAnalyzer 2100 (Agilent Technologies, USA) before cDNA library construction. The cDNA libraries were constructed using the NEBNext Ultra RNA Library Prep Kit for Illumina (NEB, USA). Illumina paired-end adapters and barcode sequences were ligated onto the cDNA fragments. The quality and quantity of the libraries were then assessed, before being sequenced using the Illumina HiSeq2000 platform (Illumina, USA). Quality control (QC) of raw reads were done using FastQC (http://www.bioinformatics.babraham.ac.uk/projects/fastqc/) and the adapters were trimmed using FastP (Chen et al., 2018) (see also Supplementary Figure 1). Reads after QC were assembled using Trinity (version 2014-07-17) (Grabherr et al., 2011). The assembled RNA-seq reads were then used as inputs for gene prediction, which were done using BRAKER (Hoff et al., 2015) and MAKER (Cantarel et al., 2008).

### Gene Prediction and Functional Annotation

EVidenceModeler (Haas et al., 2008) was used to combine the set of gene predictions from AUGUSTUS, BRAKER, and MAKER, where the gene models from the three programs were equally weighted. OrthoFinder (Emms and Kelly, 2019) was used to identify and cluster orthologous genes of the three fungal strains, and their putative functions were assessed using UniProt (The UniProt Consortium, 2019), InterProScan (Jones et al., 2014), and CAZy databases (Cantarel et al., 2009). We also performed additional manual reciprocal BLAST to find the best hits between the strains, to refine the orthologous genes, especially within the multi-copy gene clusters. We set stringent criteria for assigning a gene function to an orthologous group as follows: 1) when the genes from at least 2 out of 3 strains were assigned to the same function; 2) when none of the hits from the 3 strains agreed, we took the one with the highest BLAST score as the representative function of the orthologous group with (Supplementary Table 5).

For the predicted proteins, BLASTP (Wheeler et al., 2006) was used to align the protein sequences against the UniProtKB database Release 2019_08, using E-value cut off = 1e-6. Putative protein functions were also obtained using InterproScan (Jones et al., 2014), by matching amino acid sequences against Pfam (Finn et al., 2014), TIGRFAMs (Haft et al., 2012), SMART (Letunic et al., 2015), Prosite (Sigrist et al., 2012), and Gene3D (Lewis et al., 2018). CAZYmes Analysis Toolkit (CAT) (Cantarel et al., 2009) was employed to assess the enzymatic activities based on the similarity of amino acid sequences and or protein domain structures. Identification of enzyme active sites and protein domain was performed using HMMER 3.0 (Potter et al., 2018) (http://hmmer.org). To further assess the functional genes in biological pathways, KOALA (KEGG Orthology And Links Annotation, Kanehisa et al., 2016) was also used. Transcription levels were computed separately for each strain based on the newly assembled genomes and gene annotations. HISAT2 (Kim et al., 2019) and StringTie (Pertea et al., 2016) were used to estimate the transcript per million (TPM) values, as previously described (Cortijo et al., 2017).

### Phylogenetic Tree Reconstruction

To investigate the phylogenetic relationship of the *Aspergillus* strains based on their housekeeping (translation elongation factor 1 alpha, ubiquitin conjugating enzyme and beta-tubulin) and cellulolytic genes (AA9, GH5, 6, and 7), we constructed phylogenetic trees using the MEGA X software (Kumar et al., 2018). The evolutionary history of the genes was inferred using the maximum likelihood method with a bootstrapping test of 1,000 replicates.

## Data Availability Statement

The datasets presented in this study can be found in online repositories. The names of the repository/repositories and accession number(s) can be found at: https://www.ncbi.nlm.nih.gov/sra, PRJNA659152; https://www.ncbi.nlm.nih.gov/geo/, GSE157700.

## Author Contributions

LE, VCham, VChar, and DC conceived the overall study. AP, WP, ST, CS, and SS performed the experiments. WM and SC analyzed the data. VChar and DC wrote the manuscript with input from all authors. All authors read and approved the final manuscript.

## Conflict of Interest

The authors declare that the research was conducted in the absence of any commercial or financial relationships that could be construed as a potential conflict of interest.
