# Supplementary material for: High Quality Aspergillus aculeatus Genomes and Transcriptomes: A Platform for Cellulase Activity Optimization Toward Industrial Applications
Source: Front Bioeng Biotechnol. 2021 Jan 27;8:607176. doi: 10.3389/fbioe.2020.607176 (PMC7873481; doi:10.3389/fbioe.2020.607176)
Supplement: Supplementary file 1 [file Data_Sheet_1.pdf]

Supplementary Materials

Figure S1. A flow chart of sequencing, assembly, annotation, and analysis process for *A. aculeatus* genome and transcriptome

Figure S2. Bar graphs showing genome quantity and quality of the studies *A. aculeatus* in comparison to the established genomes of *A. niger* CBS513.88 and *A. aculeatus* ATCC16872

Figure S3. A bar graph showed the percentage of annotated genes commonly found in the three *A. aculeatus* strains.

Figure S4. Classification of Acu12065_1 (endo-1,4- glucanase) protein domain analysis by InterPro database. A predicted signal peptide (1-17 amino acids) and conserved catalytic domain (GH5, L157-H166 amino acids) with one active site, E164, were found.

Figure S5. Sequence variations between the model *A. aculeatus* strains - e.g. multiple alignments, SNP positions in relation to functional genomic positions of glycoside hydrolase family 6 (highlighted in red), cellulose-binding domain (blue), and active sites (shown by pink squares), predicted by HMMER 3.0 (Potter et. al., 2018).

Figure S6. Sequence variations between the model *A. aculeatus* strains - e.g. multiple alignments, SNP positions in relation to functional genomic positions of glycoside hydrolase family 7 (highlighted in red), cellulose-binding domain (blue), and active sites (shown by pink squares), predicted by HMMER 3.0 (Potter et. al., 2018).

Table S1. Comparison of the completeness of genome among 4 *A, aculeatus* strains *and* other 6 *Aspergillus* species using fungal_odb10 in BUSCO4

Table S2. Genome annotation and features after integrating the gene models from genome and transcriptomic data

Table S3. Statistic of transcriptome data from all replicates

Table S4. Gene numbers related to plant-based polysaccharides degradation

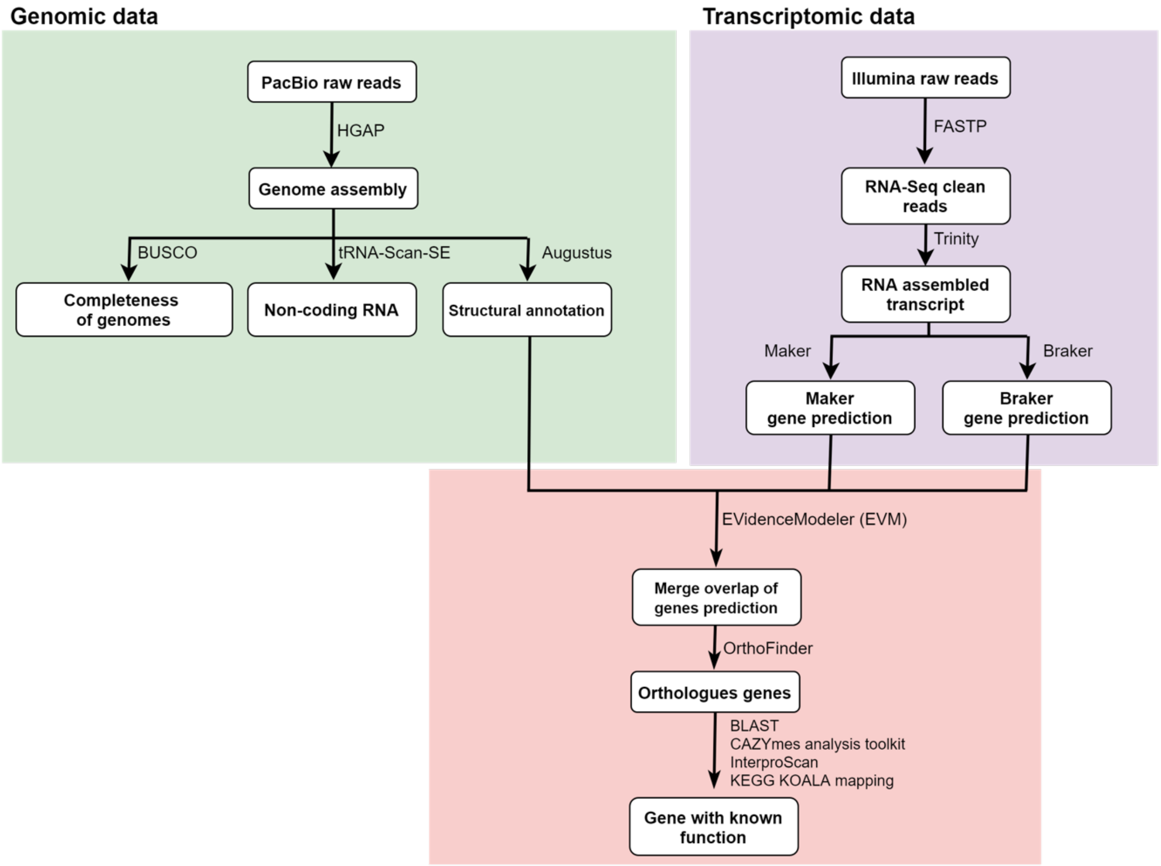

Figure S1. A flow chart of sequencing, assembly, annotation, and analysis process for *A. aculeatus* genome and transcriptome.

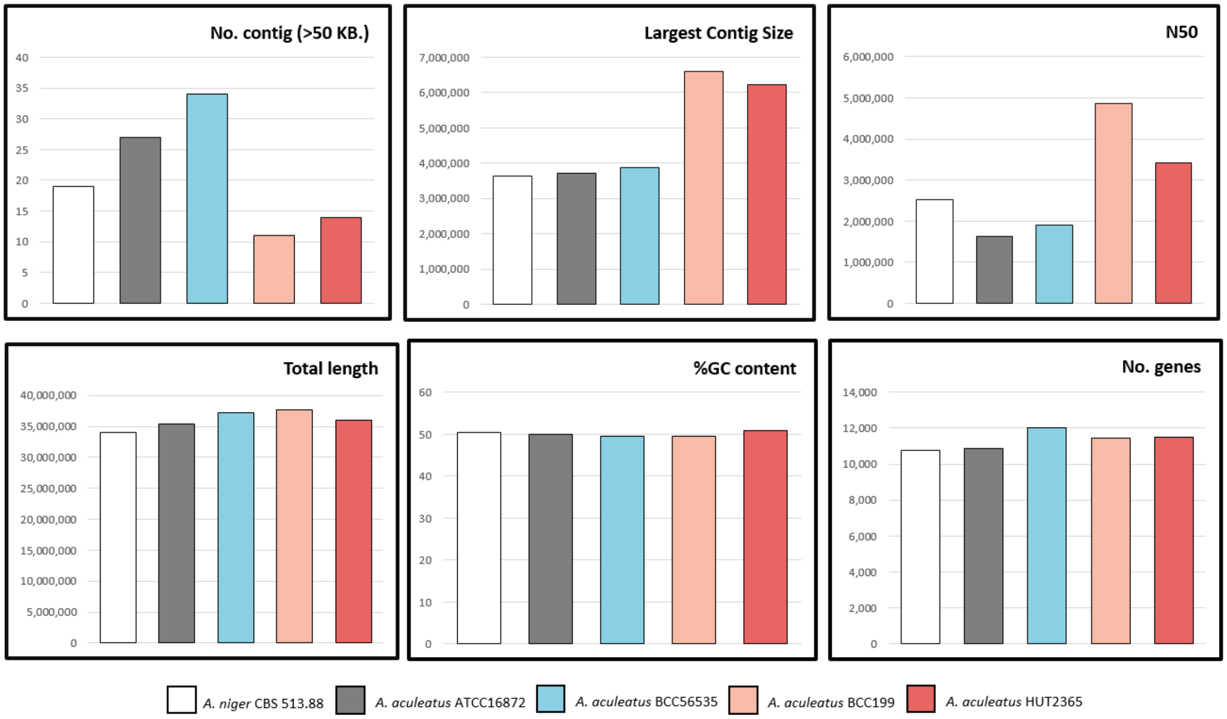

Figure S2 Bar graphs showing genome quantity and quality of the *A. aculeatus* genomes in comparison to closely-related publicly available genomes of *A. niger* CBS513.88 and *A. aculeatus* ATCC16872.

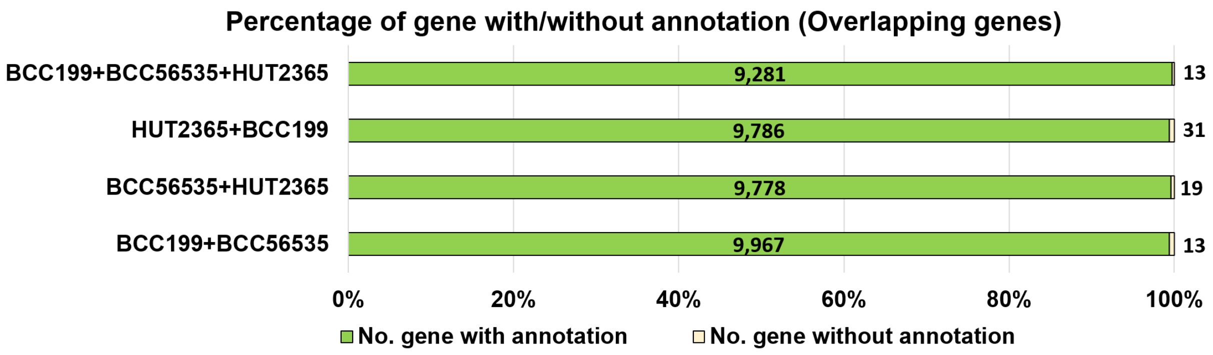

Figure S3 Bar graphs showing the percentage of annotated genes commonly found in the three *A. aculeatus* strains.

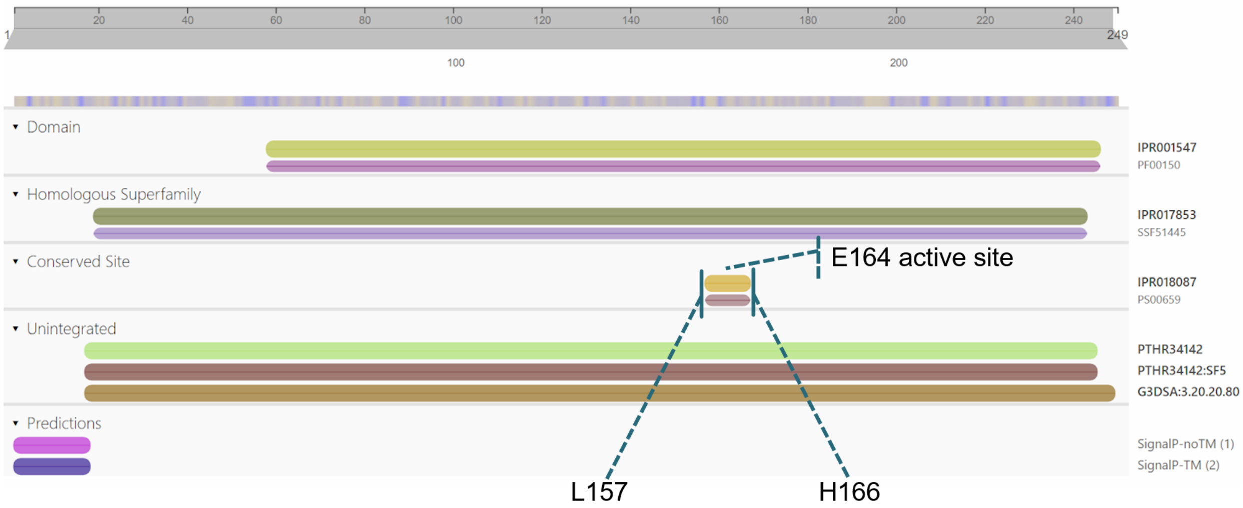

Figure S4. Classification of Acu12065_1 (endo-1,4- glucanase) protein domain analysis by InterPro. A predicted signal peptide (1-17 amino acids) and conserved catalytic domain (GH5, L157-H166 amino acids) with one active site, E164, were found.

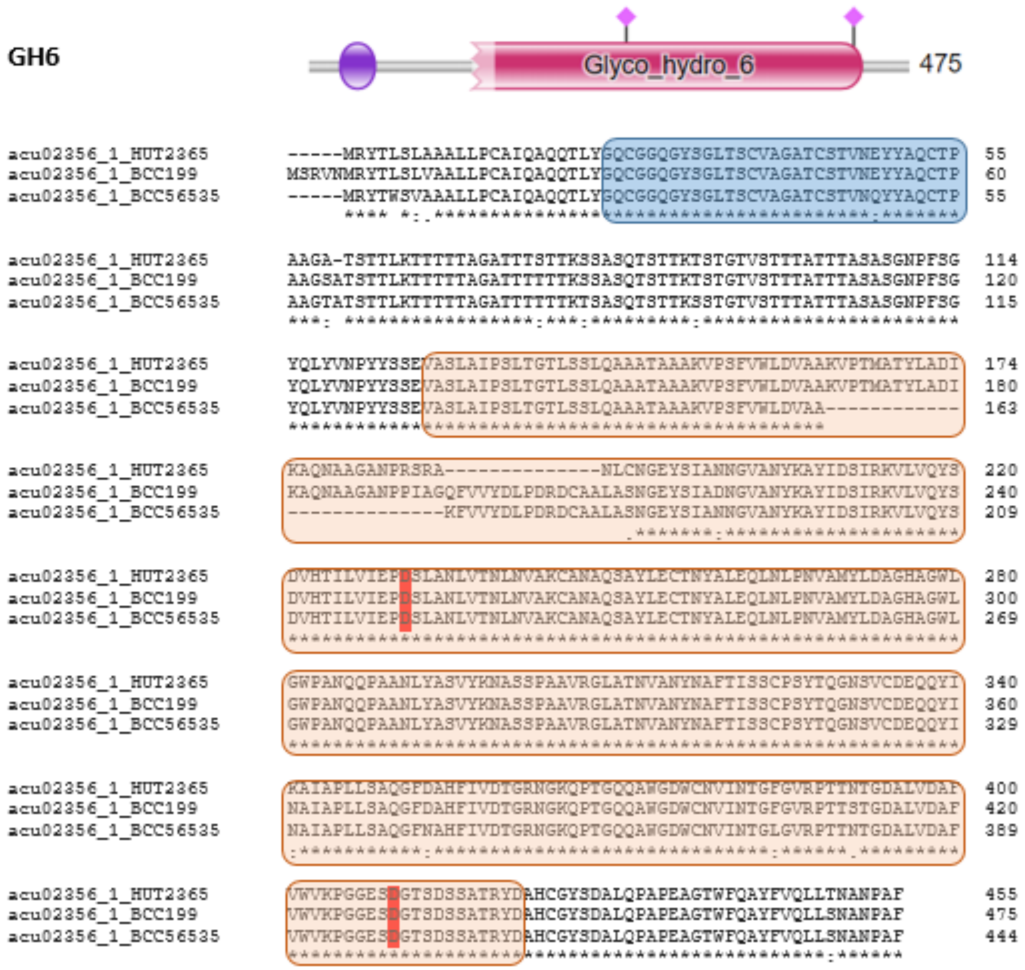

Figure S5. Sequence variations between the model *A. aculeatus* strains - e.g. multiple sequence alignments, SNP positions in relation to functional genomic positions of glycoside hydrolase family 6 (highlighted in red), cellulose-binding domain (blue), and active sites (shown by pink squares), predicted by HMMER 3.0 (Potter et. al., 2018).

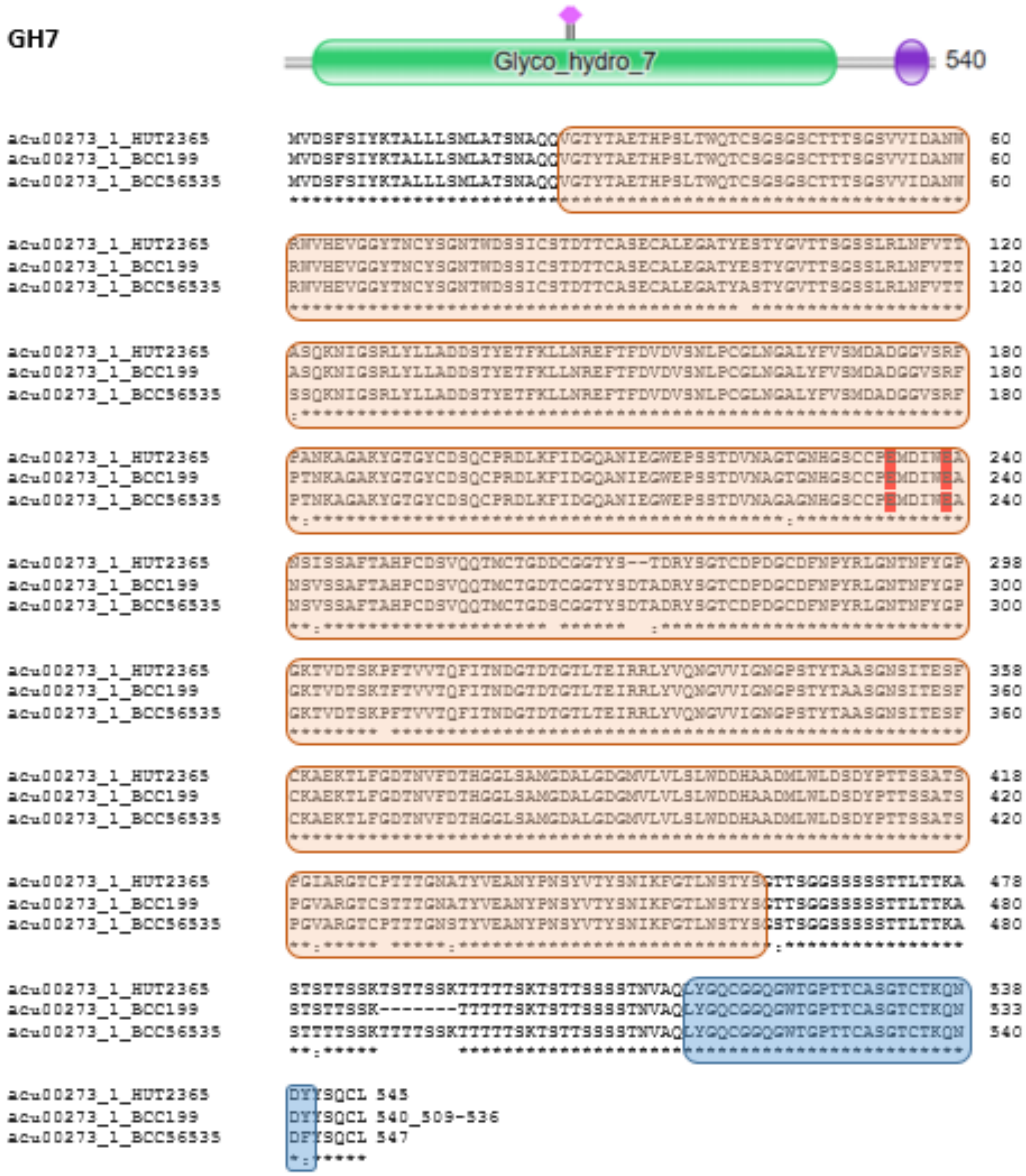

Figure S6. Sequence variations between the model *A. aculeatus* strains - e.g. multiple sequence alignments, amino acid positions in relation to functional genomic positions of glycoside hydrolase family 7 (highlighted in red), cellulose-binding domain (blue), and active sites (shown by pink squares), predicted by HMMER 3.0 (Potter et. al., 2018).

Table S1 Comparison of the functional completeness of genome among 4 *A. aculeatus* strains and other 6 *Aspergillus* species using fungal_odb10 in BUSCO4

Species	BUSCO parameters				
	Complete	Complete and single-copy	Complete and duplicated	Fragmented	Missing
A. aculeatus BCC199	96.7%	96.6%	0.1%	0.8%	2.5%
A. aculeatus BCC56535	89.7%	89.6%	0.1%	5.3%	5.0%
A. aculeatus HUT2365	92.5%	92.1%	0.4%	3.6%	3.9%
A. aculeatus ATCC16872	99.5%	99.2%	0.3%	0.1%	0.4%
A. flavus NRRL3357	93.0%	92.7%	0.3%	3.0%	4.0%
A. fumigatus AF293	98.5%	98.4%	0.1%	0.9%	0.6%
A. nidulan FGSC A4	94.0%	93.9%	0.1%	2.5%	3.5%
A. niger CBS 513.88	96.9%	95.8%	1.1%	1.1%	2.0%
A. oryzae RIB40	93.0%	92.7%	0.3%	1.6%	5.4%
A. terrius NIH2624	93.3%	93.0%	0.3%	2.5%	4.2%

Table S2 Genome annotation and features after integrating gene models from genome and transcriptomic data

Genome statistics	BCC56535	BCC199	HUT2365
Total length	37,193,364	37,670,955	36,004,422
No. Genes	12,005	11,442	11,489
Gene density (genes/Mb)	323	304	319
Age. gene length (bp)	1,547	1,615	1,555
Avg. protein length (aa)	442	476	453
Avg. exon frequency (exons/gene)	3.35	3.09	3.19
Avg. intron frequency (intron/gene)	2.35	2.09	2.19
% genes with intron	84.73%	80.00%	82.73%
% gene with Uniport Blast hit	97.11%	97.37%	97.74%

Genome statistics	BCC56535	BCC199	HUT2365
% gene annotation with enzymes (E.C. number)	16.88%	17.55%	17.40%
% gene with GO annotation	67.30%	67.33%	68.20%
% gene with CAZy	3.47%	3.75%	3.70%
Protein with signal peptide	979	1,007	975

Table S3 Statistic of transcriptome data from all replicates

Transcriptome statistics	No. of raw reads	No. of clean reads	Q20	GC content
BCC56535_lot1_A1	51.95	51.26	97.52%	55.56%
BCC56535_lot1_A2	69.56	68.38	97.39%	55.66%
BCC56535_lot2_B	45.24	44.24	96.72%	55.75%
BCC56535_lot2_C	45.89	45.21	97.40%	55.79%
BCC56535_lot2_D	49.32	47.99	96.34%	55.84%
BCC199_lot1_A1	44.43	43.33	97.28%	55.15%
BCC199_lot1_A2	71.25	70.10	97.44%	55.62%
BCC199_lot2_B	51.28	49.98	96.32%	55.79%
BCC199_lot2_C	44.22	41.51	95.12%	55.84%
HUT2365_lot1_A	68.75	67.11	97.11%	54.05%
HUT2365_lot2_B	90.78	88.92	96.67%	54.99%
HUT2365_lot2_C	55.31	54.50	97.46%	54.98%

Table S4 Gene numbers related to plant polysaccharides degradation

Polysaccharides	CAZy families	BCC56535	BCC199	HUT2365
Cellulose	GH5	12	11	11
	GH6	1	1	1
	GH7	2	2	2
	AA9	5	6	5
	Total	20	20	19
Xylan	GH10	2	2	2
	GH11	2	3	2
	GH62	1	1	1
	GH67	1	1	1
	CE1	1	1	1
	Total	7	8	7
Galactomannan	GH27	4	3	4
	GH36	2	2	2
	Total	6	5	6
Xyloglucan	GH12	3	4	4
	GH29	1	1	1
	GH74	1	1	1
	GH95	3	3	2
	Total	8	9	8
Pectin	GH28	19	19	19
	GH53	1	1	1
	GH78	7	7	6
	GH88	1	0	1

Polysaccharides	CAZy families	BCC56535	BCC199	HUT2365
	GH93	2	2	2
	GH105	2	2	2
	PL1	6	6	6
	PL4	3	3	3
	CE8	4	3	4
	CE12	3	3	2
	Total	48	46	46
Starch	GH13	15	17	16
	GH15	3	3	3
	GH31	8	7	8
	GH133	1	1	1
	Total	27	28	28
Inulin	GH32	2	2	2
